# *Streptococcus pneumoniae* Otitis Media Pathogenesis and How It Informs Our Understanding of Vaccine Strategies

**DOI:** 10.1007/s40136-017-0152-6

**Published:** 2017-05-20

**Authors:** Caroline Bergenfelz, Anders P Hakansson

**Affiliations:** Division of Experimental Infection Medicine, Department of Translational Medicine, Wallenberg Laboratory, Lund University, Inga Marie Nilsson’s Street 53, 20502 Malmö, SE Sweden

**Keywords:** Otitis media, Streptococcus pneumonia, Biofilm, Microbiome, Vaccine

## Abstract

**Purpose of Review:**

This study aimed to review the literature regarding the mechanisms of transition from asymptomatic colonization to induction of otitis media and how the insight into the pathogenesis of otitis media has the potential to help design future otitis media-directed vaccines.

**Recent Findings:**

Respiratory viruses have long been shown to predispose individuals to bacterial respiratory infections, such as otitis media. Recent information suggests that *Streptococcus pneumoniae*, which colonize the nasopharynx asymptomatically, can sense potentially “threatening” changes in the nasopharyngeal environment caused by virus infection by upregulating specific sets of genes involved in biofilm release, dissemination from the nasopharynx to other sites, and protection against the host immune system. Furthermore, an understanding of the transcriptional and proteomic changes occurring in bacteria during transition to infection has led to identification of novel vaccine targets that are disease-specific and will not affect asymptomatic colonization. This approach will avoid major changes in the delicate balance of microorganisms in the respiratory tract microbiome due to elimination of *S. pneumoniae*.

**Summary:**

Our recent findings are reviewed in the context of the current literature on the epidemiology and pathogenesis of otitis media. We also discuss how other otopathogens, such as *Haemophilus influenzae* and *Moraxella catarrhalis*, as well as the normal respiratory microbiome, can modulate the ability of pneumococci to cause infection. Furthermore, the unsatisfactory protection offered by the pneumococcal conjugate vaccines is highlighted and we review potential future strategies emerging to confer a more specific protection against otitis media.

## Otitis Media: Definitions and Complications

Otitis media (OM) comprises a heterogeneous group of inflammatory disorders affecting the middle ear (ME). With over 700 million cases annually, OM is the most common reason for pediatric emergency room visits worldwide [1–3]. By the age of three, approximately 80% of all children have experienced at least one episode of OM, while a large percentage have had three or more, due to recurrent infection [4].

OM presents itself in several subtypes with acute or chronic elements that are classified based on symptoms (such as fever, irritability, pulling of the ear); visual appearance and/or perforation of the tympanic membrane; and presence of ME fluid, with and without active inflammation [5]. Acute otitis media (AOM) generally affect children under the age of two and is characterized with sudden onset of symptoms, significant pain, and signs of inflammation with accumulation of purulent fluid behind the tympanic membrane. Based on new directives in several countries, including Sweden and parts of the USA, AOM in otherwise healthy children is usually treated with general analgesia, antipyretic drugs, and only for severe and recurrent cases with antibiotics. Despite this, OM is still a major reason for outpatient antibiotic prescription in the USA [2] and in many other parts of the world.

In contrast, secretory otitis media (SOM, sometimes referred to as otitis media with effusion) is defined as a chronic inflammatory condition mainly affecting children between the age of 3 and 7. SOM may occur as a sequela to AOM and is generally not associated with any signs of an acute infection but presents with fluid behind the tympanic membrane and is associated with hearing impairment that over time can lead to cognitive and developmental problems [6••, 7]. The continuum of manifestations, ranging from uncomplicated, asymptomatic and self-limiting conditions to life-threatening, recurrent or chronic disorders with associated severe sequelae such as deafness, acute mastoiditis, or cholesteatoma are likely explained by the multifactorial and polymicrobial nature of OM [6••].

### The Microbiology of Otitis Media

Respiratory viruses such as influenza viruses (A and B), rhinoviruses, respiratory syncytial virus (RSV), and adenoviruses are well-known causes of both asymptomatic and symptomatic OM [8, 9]. Besides causing infection on their own, viruses commonly predisposes individuals to bacterial AOM that is generally more symptomatic and primarily caused by the bacterial triad *Streptococcus pneumoniae* (the pneumococcus), *Haemophilus influenzae*, and *Moraxella catarrhalis* [10]. A fourth pathogen worth mentioning is *S. pyogenes*, which although it only causes a small percentage of AOM cases, is the second most common organism associated with AOM complications [11]. The same bacterial species are also detected in ME fluid from patients with SOM, although an increased occurrence of bacteria such as *Pseudomonas aeruginosa* and *S. aureus* are detected in the ME fluid of these patients [6••, 12–14]. Whether this change in species tropism is indicative of a change in the ME environment, as the acute infection transitions into a more chronic state, or suggests that AOM and SOM are two separate clinical entities is currently not completely understood. Even though there are bacterial strains that are more otogenic, the bacteria associated with various subtypes of OM mostly reflect the composition of the normal nasopharyngeal microflora, and disease is associated with changes in the host environment that provides an opportunity for these organisms to actively move to the site of infection [15]. Among these bacterial organisms, *S. pneumoniae* and *H. influenzae* are the most common causes of OM overall, regardless of subtype.

*S. pneumoniae*, the focus of this review, accounts for 30–50% of all AOM cases in different parts of the world, which amounts to approximately 300 million cases annually [1, 16••, 17, 18]. *S. pneumoniae* is also an especially important pathogen in OM, as it is the main cause of recurrent infections as well as postinfectious complications, including ventilation tube insertion (myringostomy) [19••, 20••].

## Epidemiologic Correlations Between Colonization and Disease

Although pneumococcal colonization is widespread, and mostly asymptomatic, transition to disease occurs frequently enough to make *S. pneumoniae* one of the main causes of respiratory tract infections, such as OM, sinusitis, and pneumonia worldwide [3, 21–25].

### Bacterial Colonization and Otitis Media

*S. pneumoniae* effectively colonize the mucosal surfaces of the nasopharynx (NP) beginning within the first few weeks or months of life [26••]. It is commonplace for children to be successively colonized and by the time they reach the age of two, greater than 95% of children will have been colonized with individual serotypes for weeks or months that are sequentially replaced as more serotypes are acquired [27–29]. The frequency and time of colonization in the NP has long been associated with increased risk of OM [30, 31] Furthermore, otitis-prone children are more heavily colonized than are non-otitis-prone children [10, 30, 32, 33], suggesting that the resident normal microflora participates in the pathogenesis of OM. Indeed, it is well established that colonization of the human NP always precedes the dissemination of bacteria to other sites, such as the ME, sinuses, lungs, as well as meninges and blood [3, 21, 34].

### Role of Viral-Bacterial Interactions in Induction of Pneumococcal Otitis Media

Epidemiologically, transition from NP colonization to OM is highly correlated with concomitant viral infection [8, 35, 36]. Indeed, many animal models of infection require or augment bacterial infection in the presence of a preceding viral infection [10, 37–39]. Essentially all upper respiratory tract viruses can predispose secondary bacterial OM, albeit with different propensities [10]. The mechanisms underlying this predisposition are in generally associated with suppression of the host immune response, although bacterial factors may play a partial role as well. Additionally, other changes to the host NP environment or co-colonization with other species of the respiratory tract microflora, such as the potential otopathogens *H. influenzae* and *M. catarrhalis* [30, 40–46], or the normal microflora are common and can modulate the pneumococcal transition from colonization to disease [26••, 47, 48••] (see below for more detail).

## Transition from Asymptomatic Biofilm Colonization to Otitis Media

### Presence of Biofilms During Colonization and Disease

It has been speculated over the years that pneumococci colonizing the NP may form biofilms [49–53], but it was not until recently that this was shown in mice in vivo [54••, 55, 56]. Biofilms are collaborating multicellular communities closely associated with the mucosal epithelium and encased in a self-produced polymeric matrix that often incorporates host components [57]. Aggregation in sessile biofilms provide the bacteria with a survival advantage in the harsh NP environment that is devoid of ample nutrition and where bacteria are exposed to factors of the innate immune response. [58–60]. As biofilms are inherently more resistant to antibacterial agents and able to resist host-immune responses, this will ultimately facilitate persistence and support the dissemination of virulent clones in the population.

Biofilms are present in up to 80% of all infections and are typically found in chronic and recurrent infections, such as OM [61]. Prior to our observation that asymptomatically colonizing pneumococci grow on the mucosal surface as biofilms [54••, 55], in vivo biofilm formation had only been shown during disease states in association with adenoids and mucosal epithelium of children with recurrent or chronic ME disease and chronic rhinosinusitis [62••, 63, 64, 65, 66]. However, it is unclear whether the biofilms detected at these disease sites represent asymptomatic colonization and persistence of bacteria from which virulent bacteria may seed off under the right conditions or if they are directly involved in the disease process [50, 53, 67].

### Mechanisms of Transition to Otitis Media

Biofilm bacteria grown in vitro have an avirulent phenotype and are unable to cause AOM in animals [53, 68, 69••]. This is true also when bacterial biofilm colonization is induced in animals in vivo [69••]. However, virus infection is known to increase adherence of bacteria to epithelial cells [70–73], compromise the function of the Eustachian tube, induce dysfunction of the mucosal epithelium and immune cells [10], as well as to induce inflammation, fever, and the release of cytokines and other “danger signals” in the secretion [74–76]. All of these factors potentially contribute to the disruption of colonization and dissemination of bacteria.

Indeed, pneumococci are able to sense alterations in the NP environment resulting from virus infection [69••], including ATP and glucose release from damaged tissue, norepinephrine release from activation of a sympathomimetic response, and increased temperature associated with fever [77–80]. Each of these stimuli was shown to induce dispersal and dissemination of bacteria from asymptomatically colonized mice, resulting in severe AOM [69••, 81]. The increased virulence of biofilm-dispersed bacteria in various animal infection models, including AOM, compared to planktonic, broth-grown bacteria, is explained by the major differences between the transcriptomes of the bacterial populations. Dispersed bacteria produced from an in vitro model system [82] exhibited major changes in their transcriptome with 134-1179 genes (depending on dispersal stimuli) being significantly up- or downregulated when compared to biofilm bacteria [83••]. Genes associated with carbohydrate metabolism, bacteriocin production, and common virulence factors such as capsule, *pspA*, *ply*, *pcpA*, *nanA*, and *nanB* were upregulated in dispersed bacteria, whereas competence genes and adhesins were downregulated [83••]. These results are supported by a proteomic study by Hall-Stoodley’s laboratory, who showed that biofilm bacteria use alternative metabolic pathways and downregulate capsule and other virulence factors when compared to broth-grown bacteria [84••]. These results have provided a better understanding of the mechanisms involved in the induction of otitis media and have explained the increased virulence seen in animals infected with actively released biofilm bacteria compared to broth-grown bacteria.

### Modulation of Disease by the Respiratory Tract Microbiome

Additionally, there is evidence that the normal microbiome is involved in modulating the transition from colonization to infection. In an elegant study by Bogaert and coworkers, children were followed for 12 months and the microbiome in the nasopharynx was analyzed and compared with the incidence of respiratory illnesses. In this study, bacterial species from the normal flora, such as *Dolosogranulum spp*., *Corynebacterium spp*., and *Moraxella spp*., were associated with less AOM, whereas *Veillonella spp.* and high levels of *S. pneumoniae* and *H. influenzae* were associated with a higher incidence of infection [26••]. The exact role of the interaction between *S. pneumoniae* and *H. influenzae* in vivo is not clear as studies have shown that non-typeable *H. influenzae* promoted pneumococcal biofilm formation in vitro [85], as well as in the chinchilla model of AOM, and reduced the incidence of systemic disease [67]. The effect on local infection is unclear.

Studies by Pettigrew and coworkers have, similarly, shown that *Corynebacterium* and *Dolosigranulum* are protective for development of OM, and that *Actinomyces*, *Rothia*, *Neisseria*, and *Veillonella* are associated with an increased risk for development of AOM [48••]. In contrast to Biesbroek et al., this study showed an association between *Moraxella spp*. and an increased colonization and risk for AOM with *S. pneumoniae*. This association is supported by studies showing that the presence of *M. catarrhalis* is a marker for increased severity of infection with other pathogens, including *S. pneumoniae*, and corresponding increases in the risk of AOM [86, 87]. Additionally, the ascension of pneumococci into the ME of mice was increased in the presence of *M. catarrhalis* in the nasopharynx, and *M. catarrhalis* was able to passively protect pneumococci against β-lactam killing in dual biofilms [88]. Still, the role of *Moraxella spp.* in disease induction could potentially differ with age as the ages of the study participants was lower in the Biesbroek study than in the Laufer study, and the protection against respiratory illnesses was especially pronounced at an early age. Based on this information, no clear picture of the role of the interaction between various species present in the nasopharynx and their role in protection or induction of AOM can be found.

## Vaccines

### Effectiveness of Pneumococcal Conjugate Vaccines Against Otitis Media

The poor immunogenicity of the 23-valent polysaccharide vaccine in infants and young children resulted in the development of the 7-valent pneumococcal conjugate vaccine Prevnar® (PCV7), released in 2000. This vaccine showed a strong immunogenicity in children [89]. Over the years, vaccines with increased valency have been developed to be more broadly protective and more suitable for the serotype distribution responsible for disease in other places than the USA. As of September 2016, conjugate vaccines, including Synflorix® (PHiD-CV or PCV10), a 10-valent pneumococcal vaccine with *H. influenzae* protein D, and PCV13 have, together with PCV7, been implemented in 132 countries around the world [90]. The vaccine efficacy has been most striking for invasive pneumococcal disease (IPD) with up to 93% protection detected [91••]. The effects of the pneumococcal conjugate vaccines (PVCs) against OM and its complications have unfortunately not provided similar levels of protection [91••, 92, 93••, 94, 95].

Now, several years after the introduction of the PCV vaccines, it has become apparent that none of the vaccines have had a significant impact on nasopharyngeal colonization with *S. pneumoniae* [92, 93••, 96–98]. This is despite the 55–77% reduction in vaccine serotype-induced AOM compared to the prevaccine era [16••, 91••] and the 35–46% reduction in all pneumococcal AOM [91••, 99, 100]. This suggests that the vaccines have eliminated colonization with most vaccine serotypes but that serotype replacement with non-vaccine types has occurred rather immediately. However, these studies also indicate that the non-vaccine serotypes establishing colonization in the nasopharynx are, at least so far, less able to cause AOM. Interestingly, the all-cause AOM is estimated to be reduced by only 0–7% in many regions of the world [91••, 101, 102], despite a reduction in pneumococcal OM, which suggest both that a concomitant increase in serotype replacement by non-vaccine serotypes is taking place to some extent and that AOM caused by other organisms is increasing. However, with the introduction of PCV13, some parts of the world with high levels of pneumococcal colonization and disease have seen a 24–68% reduction in all-cause AOM [16••, 103, 104], suggesting an increased efficacy of the vaccine in areas with the highest disease burden. As not enough time has passed to make long-term predictions about the efficacy of these vaccines, continued monitoring of the nasopharyngeal microflora and causes of AOM will be important in the future in various parts of the world.

Although the total effect on pneumococcal AOM has not been as promising as for invasive disease, a major benefit with the current vaccines is that a 10–26% reduction in recurrent AOM and a 24–32% reduction in severe and complicated AOM, including a reduction of ventilation tube insertion, have been observed [19••, 105]. This is a positive result, albeit not completely surprising, as *S. pneumoniae* is most often associated with increased recurrence and severity of infection [20••, 106].

### Future Vaccine Strategies

Based on the incomplete protection against *S. pneumoniae*-induced AOM conferred by the conjugate vaccines, and the fact that the evolutionary pressure and other causes will make the current vaccines less efficacious over time, several investigators have indicated that novel approaches, alone and in combination with current vaccine strategies, need to be implemented in the near future [94, 107••, 108]. The fact that natural protection against pneumococcal colonization is dependent on antibodies to pneumococcal proteins, rather than capsular polysaccharide, suggests that an approach involving protein vaccine candidates may be most fruitful [109]. Whole-cell vaccines and protein vaccines with broad coverage across the over 95 serotypes are two approaches that have been suggested and investigated [94, 107••]. AOM-specific antigen targets have been identified in clinical studies of convalescent sera from AOM patients. Alpha-enolase, streptococcal lipoprotein rotamase A, putative proteinase maturation protein, histidine triad protein D, pneumolysin, pneumococcal surface adhesin A (PsaA), and pneumococcal surface protein A (PspA) are potential proteins that could be used for AOM-specific vaccine protection in the future [110–113].

Experiments with PspA have shown that the presence of anti-PspA antibodies in children reduces the risk of AOM [114]. Furthermore, mucosal immunization can protect mice against virus-induced AOM and reduces colonization burden of pneumococci [115, 116]. In studies by Xu et al., PsaA was shown to protect mice from AOM [117]. Similarly, a study by Tuomanen and coworkers has shown that a fusion protein of pneumolysin and choline binding protein A (CbpA, PspC) protected against OM in mice [118]. It will be interesting to follow the development of these strategies as and if they progress towards finished vaccines.

### Potential Disease-Specific Vaccines Against AOM

Each of the antigens mentioned above resulted in that the colonization burden was reduced concomitant to AOM protection. The advantage of eliminating pneumococcal colonization is that it will have a higher efficacy on the population as a whole, even with more targeted immunization policies, due to herd immunity. On the other hand, *S. pneumoniae* has evolved to become a harmless commensal organism in the nasopharyngeal microflora that inhibits the expansion of potentially more harmful organisms such as *S. pyogenes* and *S. aureus* through competitive exclusion [119, 120]. The elimination of *S. pneumoniae* from this niche may therefore change the nasopharyngeal microbiome in a way that may or may not be detrimental in the future.

As an alternative, we have recently used the information gained from our studies regarding the transition from colonization to infection by choosing antigens that are conserved and highly upregulated on the surface of disease-causing organisms (i.e., the biofilm-dispersed pneumococci described above) [83]. In two studies, we have used either PspA [113] or eight antigens chosen for their upregulation in disease-causing organisms [121] and immunized mice systemically rather than mucosally, to avoid effects on asymptomatic colonization. In both studies, we could protect mice from AOM with several antigens and against a panel of strains of different serotypes present and absent in the current vaccines, without affecting NP colonization [113, 121]. This approach may provide one avenue towards disease-specific pneumococcal vaccines that avoid affecting the delicate balance between microorganisms in the nasopharynx.

## Concluding Remarks

The fact that pneumococci grow as biofilms in the murine NP during asymptomatic carriage [54••] has changed the way we consider both colonization and potential transition to infection. Bacteria within biofilms formed during NP colonization must balance attachment, growth, and eventual dispersion processes within a dynamic NP environment. Of these three critical steps, biofilm dispersal is by far the least understood. For *S. pneumoniae*, this is particularly troubling as biofilm dispersion represents the main step associated with clinical infections in susceptible patients: it is precisely these detached cells that are primed for dissemination to normally uninfected sites and are the causative agents of OM. Future studies are needed to better understand the exact mechanism of transition from asymptomatic colonization to infection and the molecules involved in pathogenesis in the disseminated and virulent population. Results from such studies have the potential to result in novel approaches to interfere with disease induction to prevent AOM and could be especially important for otitis-prone children.

The conjugate vaccines have had an enormous impact on IPD, for which the vaccines were designed, but have had a much lower efficacy against pneumonia and OM. The response to some of the serotypes, including 23F and 6B, is known to be lower based on poorer immunogenicity [122], and vaccine escape is easily observable for the vaccine-serotypes 19F, 23F, and 6B in AOM cases [101, 123, 124]. As new formulations of the conjugate vaccines have been introduced, it is becoming clearer that to provide better protection against AOM in the future, we will need to add to the current vaccine regimen using protein vaccines with broader and more AOM-specific activities. An understanding of the pathogenesis of AOM and the bacterial changes associated with virulence has great potential to provide clues to future effective vaccine targets.
